# Predictive value of the C-reactive protein/albumin ratio in severity and prognosis of acute pancreatitis

**DOI:** 10.3389/fsurg.2022.1026604

**Published:** 2023-01-10

**Authors:** Yi Zhao, Wenwen Xia, You Lu, Wei Chen, Yan Zhao, Yugang Zhuang

**Affiliations:** ^1^ Department of Emergency, Shanghai Tenth People’s Hospital of Tongji University, Shanghai, China; ^2^ Department of Gastroenterology, Shanghai Tenth People’s Hospital of Tongji University, Shanghai, China; ^3^ Department of Respiratory Medicine, Shanghai Tenth People’s Hospital of Tongji University, Shanghai, China

**Keywords:** c-reactive protein/albumin ratio, Ranson score, MCTSI score, BISAP score, prognosis, acute pancreatitis

## Abstract

**Aim:**

To investigate the predictive value of C-reactive protein (CRP) to serum albumin (ALB) ratio in the severity and prognosis of acute pancreatitis (AP), and compare the predictive value of the CRP/ALB ratio with the Ranson score, modified computed tomography severity index (MCTSI) score, and Bedside Index of Severity in Acute Pancreatitis (BISAP) score.

**Methods:**

This cohort study retrospectively analyzed clinical data of AP patients from August 2018 to August 2020 in our hospital. Logistic regression analysis was utilized to determine the effects of CRP/ALB ratio, Ranson, MCTSI, and BISAP score on severe AP (SAP), pancreatic necrosis, organ failure, and death. The predictive values of CRP/ALB ratio, Ranson, MCTSI, and BISAP score were examined with the area under the curve (AUC) of the receiver operator characteristic (ROC) curve analysis. DeLong test was used to compare the AUCs between CRP/ALB ratio, Ranson, MCTSI, and BISAP score.

**Results:**

Totally, 284 patients were included in this study, of which 35 AP patients (12.32%) developed SAP, 29 (10.21%) organ failure, 30 (10.56%) pancreatic necrosis and 11 (3.87%) died. The result revealed that CRP/ALB ratio on day 2 was associated with SAP [odds ratio (OR): 1.74, 95% confidence interval (CI): 1.32 to 2.29], death (OR: 1.73, 95%CI: 1.24 to 2.41), pancreatic necrosis (OR: 1.28, 95%CI: 1.08 to 1.50), and organ failure (OR: 1.43, 95%CI: 1.18 to 1.73) in AP patients. Similarly, CRP/ALB on day 3 was related to a higher risk of SAP (OR: 1.50, 95%CI: 1.24 to 1.81), death (OR: 1.8, 95%CI: 1.34 to 2.65), pancreatic necrosis (OR: 1.22, 95%CI: 1.04 to 1.42), and organ failure (OR: 1.21, 95%CI: 1.04 to 1.41). The predictive value of CRP/ALB ratio for pancreatic necrosis was lower than that of MCTSI, for organ failure was lower than that of Ranson and BISAP, and for death was higher than that of MCTSI.

**Conclusion:**

The CRP/ALB ratio may be a novel but promising, easily measurable, reproducible, non-invasive prognostic score that can be used to predict SAP, death, pancreatic necrosis, and organ failure in AP patients, which can be a supplement of Ranson, MCTSI, and BISAP scores.

## Introduction

Acute pancreatitis (AP) is an inflammatory disease of the pancreas characterized by acute abdominal pain and elevated serum pancreatin, which leads to subsequent pancreatic autodigestion, edema, hemorrhage, necrosis, and even distal organ dysfunction (1, 2). The majority of AP cases are mild and with an acceptable prognosis, whereas approximately 20% of AP patients develop moderate-to-severe AP (MSAP) and even severe AP (SAP), characterized by rapid progression, poor prognosis, and a high mortality rate of 30% (3). The long hospital stays and a series of financial burdens brought by SAP make us have to keep alert to the severity and poor prognosis of AP (4, 5). Early recognition of disease severity, as well as early evaluation of prognostic factors, may be helpful for early therapeutic intervention so as to improve survival and prognosis.

At present, a variety of scores are used in the assessment of AP prognoses, such as Ranson, modified computed tomography severity index (MCTSI), and Bedside Index of Severity in Acute Pancreatitis (BISAP) scores (6–8). However, the calculation of those scores requires the use of numerous parameters and very complicated algorithms, which limits their use in clinical practice (8). Therefore, there is an urgent need for a simpler, faster, real-time tool to predict disease prognosis. In several studies, serum markers such as C-reactive protein (CRP) and albumin (ALB) have been reported to be associated with prognosis in AP (9–11). However, their predictive value is unsatisfactory when used alone (12). Accumulating studies reported that CRP/ALB ratio could predict the prognosis of diseases associated with inflammatory response (10, 13, 14). A study by Zhao et al. (12) demonstrated that the admission CRP/ALB ratio was significantly higher in the re-operation of AP patients under debridement. The CRP/ALB ratio may be associated with the prognosis of AP. Kaplan et al. (15) reported that the CRP/ALB ratio could predict the mortality of AP patients. However, to date, the relationship between the CRP/ALB ratio and severity of AP, such as SAP, organ failure, and pancreatic necrosis in patients with AP remains unclear. It is necessary to select a simple, non-invasive method to predict the severity of AP in order to help to improve survival.

Herein, we investigated the predictive value of the CRP/ALB ratio for the determination of severity and prognosis in patients who were with an AP diagnosis and compared the predictive value of the CRP/ALB ratio with the Ranson score, MCTSI score, and BISAP score.

## Methods

### Study design and participants

In this cohort study, we reviewed the data of AP patients in the Shanghai Tenth People's Hospital of Tongji University between August 2018 and August 2020. Totally, 284 patients were enrolled in the study. The inclusion criteria were: (1) all AP patients who were admitted to our hospital within 72 h of onset met the International Association of Pancreatology (IAP)/American Pancreatic Association (APA) guidelines (16); (2) patient was admitted to hospital within 72 h of onset, and abdominal enhanced CT examination was performed within 2–7 days of admission. The exclusion criteria were: (1) presence of malignant tumor, recent other infectious diseases, diseases of the blood system, rheumatic immune diseases, and organ dysfunction; (2) age <18 years; (3) acute onset of chronic pancreatitis (on the basis of patient history and CT imaging); (3) pregnant or lactation population; (4) combination with other digestive system diseases; (5) incomplete medical records. All procedures were implemented in accordance with the principles of the Declaration of Helsinki and the design of the work was reviewed and approved by our Ethics Committee (SHYS-IEC-5.0/22K161/P01). Once data is truly anonymized and individuals are no longer identifiable, patient consent was waived.

### Data collection

The clinical information of AP patients who met the inclusion criteria was collected from the medical record, including: (1) Information at the time of hospitalization: gender (male, female), age (years), etiology (gallstone, hyperlipidemia, alcohol, and others); (2) scoring system: Ranson score, MCTSI score, and BISAP score were evaluated for each patient, respectively; (3) laboratory indicators: white blood cell (WBC, 10^9^/L), hemoglobin (Hb, g/L), neutrophil counts (10^9^/L), lymphocyte counts (10^9^/L), platelet counts (10^9^/L), platelet to lymphocyte ratio (PLR), neutrophil to lymphocyte ratio (NLR), alanine transaminase (ALT, U/L), aspartate aminotransferase (AST, U/L), direct bilirubin (DB, umol/L), total bilirubin (TB, umol/L), gamma-glutamyl transpeptidase (GGT, U/L), alkaline phosphatase (AKP, U/L), amylase (AMY, U/L), lipase (LPS, U/L), blood urea nitrogen (BUN, mmol/L), serum creatinine (Scr, umol/L), calcium (Ca, mmol/L), and lactate dehydrogenase (LDH, U/L) after admission for all patients in this study; (4) patient outcomes: SAP, infectious pancreatic necrosis, organ failure, and death.

### Variable definition and outcome

All AP patients who were admitted to our hospital within 72 h of onset met the guidelines for the Diagnosis and Treatment of Acute Pancreatitis revised in 2019 by the Pancreatic Surgical Science Section of the Chinese Medical Association Surgery Branch, which requires at least two conditions: (1) abdominal pain highly suggestive of AP; (2) elevations in serum amylase and/or lipase to more than 3 times the upper limit of normal; (3) the presence of characteristic radiological findings [ultrasonography or computerized tomography (CT)] of AP.

The definition of severity of AP was used as follows: (1) mild AP (MAP): no organ failure and no local or systemic complications; (2) MSAP: organ failure that resolves within 48 h (transient organ failure) and/or local or systemic complications without persistent organ failure; and (3) SAP: persistent single or multiple organ failure (>48 h). Following the modified Marshall scoring system, organ failure was defined as a score of 2 or more for one of three organ systems (respiratory, renal, and cardiovascular) (17). Death was defined as death during hospitalization.

### Prognostic groups

According to the clinical outcome, all patients were divided into the death group (*n* = 11) and the survival group (*n* = 273). Patients were also divided into the SAP group (*n* = 35) and the non-SAP group (*n* = 249). According to the presence or absence of organ failure, the two groups were divided into the organ failure group (*n* = 29) and the without organ failure group (*n* = 255). Abdominal enhanced CT scan results confirmed the occurrence of pancreatic necrosis in 30 patients with pancreatitis in this study, and AP patients were divided into pancreatic necrosis group (*n* = 29) and non-pancreatic necrosis group (*n* = 255).

### Statistical analysis

Normally distributed data described with mean ± SD, an unpaired student t-test was used for comparison between groups. Non-normally distributed data defined with median and interquartile range [M (*Q*_1_, *Q*_3_)] were compared between the two groups using the Mann–Whitney *U* test. Categorical variables expressed as numbers and percentages [*n* (%)] were compared between the two groups using the *χ*^2^ test or Fisher's exact test. Differential analysis was used to screen for possible confounding factors. Univariable logistic regression and multivariate logistic regression analysis were utilized to determine the effects of CRP/ALB ratio, Ranson score, MCTSI score, and BISAP score on SAP, infectious pancreatic necrosis, organ failure, and death. The predictive values of CRP/ALB ratio, Ranson score, MCTSI score, and BISAP score on SAP, infectious pancreatic necrosis, organ failure, and death were examined with the area under the curve (AUC) of the receiver operator characteristic (ROC) curve analysis. DeLong test was used to compare the AUCs between CRP/ALB ratio, Ranson score, MCTSI score, and BISAP score. Statistical power was tested for outcome with a smaller sample size. Pairwise comparisons were performed with a Bonferroni correction for multiple comparisons. The *P* value < 0.05 was considered to be statistically significant. SAS 9.4 software (SAS Institute, Cary, NC, United States) was performed to compute the statistical analysis.

## Results

### Characteristics of patients in different outcomes groups

The study population consisted of 284 patients, 123 females (43.31%) and 161 (56.69%) males. The median age of the patients was 59.50 (IQR 39.00–70.00) years. The median hospitalization length of the patients was 9 (IQR 8–13) days. In terms of etiology, 154 (54.23%) of the patients were due to gallstone, 69 (24.30%) were due to hyperlipidemia, 13 (4.58%) due to alcohol, and 61 (21.48%) due to other etiologies (autoimmune diseases, metabolic diseases, trauma or postoperative, drugs, viral infection, etc., or the specific etiologies of pancreatitis cannot be determined). The baseline characteristics of patients are summarized in Table 1.

**Table 1:** Differences of population characteristics between patients with and without SAP.

Variables	Total (*n* = 284)	Non-SAP (*n* = 249)	SAP (*n* = 35)	Statistics	*P*
Gender, *n* (%)				*χ*^2^ = 0.003	0.954
Female	123 (43.31)	108 (43.37)	15 (42.86)		
Male	161 (56.69)	141 (56.63)	20 (57.14)		
Age, years, M (*Q*_1_, *Q*_3_)	59.50 (39.00, 70.00)	60.00 (41.00, 70.00)	57.00 (38.00, 68.00)	*Z* = −1.167	0.243
**Etiology**					
Gallstone, *n* (%)				*χ*^2^ = 0.514	0.473
No	130 (45.77)	112 (44.98)	18 (51.43)		
Yes	154 (54.23)	137 (55.02)	17 (48.57)		
Hyperlipidemia, *n* (%)				*χ*^2^ = 5.353	0.021
No	215 (75.70)	194 (77.91)	21 (60.00)		
Yes	69 (24.30)	55 (22.09)	14 (40.00)		
Alcohol, *n* (%)				–	0.206
No	271 (95.42)	239 (95.98)	32 (91.43)		
Yes	13 (4.58)	10 (4.02)	3 (8.57)		
Other, *n* (%)				*χ*^2^ = 2.391	0.122
No	223 (78.52)	192 (77.11)	31 (88.57)		
Yes	61 (21.48)	57 (22.89)	4 (11.43)		
Ranson, M (*Q*_1_, *Q*_3_)	2.00 (1.00, 3.00)	1.00 (1.00, 3.00)	5.00 (4.00, 6.00)	*Z* = 8.427	<0.001
MCTSI, M (*Q*_1_, *Q*_3_)	2.00 (2.00, 4.00)	2.00 (2.00, 4.00)	6.00 (6.00, 8.00)	*Z* = 7.953	<0.001
BISAP, M (*Q*_1_, *Q*_3_)	1.00 (0.00, 2.00)	1.00 (0.00, 1.00)	3.00 (2.00, 3.00)	*Z* = 8.327	<0.001
WBC, 10^9^/L, M (*Q*_1_, *Q*_3_)	10.48 (7.44, 14.46)	10.18 (7.30, 13.41)	16.14 (9.85, 22.39)	*Z* = 4.224	<0.001
Hb, g/L, Mean ± SD	130.70 ± 21.10	130.63 ± 20.06	131.23 ± 27.71	*t* = −0.12	0.903
Neutrophil counts 10^9/^L, M (*Q*_1_, *Q*_3_)	8.56 (5.80, 12.26)	8.29 (5.53, 11.34)	14.38 (8.22, 20.24)	*Z* = 4.502	<0.001
Lymphocyte counts, 10^9^/L, M (*Q*_1_, *Q*_3_)	1.15 (0.82, 1.48)	1.18 (0.84, 1.51)	1.00 (0.71, 1.31)	*Z* = −1.697	0.090
Platelet counts, 10^9^/L, M (*Q*_1_, *Q*_3_)	196.00 (150.50, 245.00)	194.00 (150.00, 239.00)	213.00 (158.00, 266.00)	*Z* = 1.035	0.301
PLR, M (*Q*_1_, *Q*_3_)	178.05 (119.44, 242.36)	169.90 (117.75, 236.36)	211.00 (162.89, 259.00)	*Z* = 2.074	0.038
NLR, M (*Q*_1_, *Q*_3_)	7.84 (4.67, 13.31)	6.78 (4.42, 11.60)	12.40 (8.58, 20.05)	*Z* = 4.631	<0.001
ALT, U/L, M (*Q*_1_, *Q*_3_)	42.70 (24.55, 145.20)	43.80 (25.10, 151.70)	32.50 (18.30, 83.60)	*Z* = −1.533	0.125
AST, U/L, M (*Q*_1_, *Q*_3_)	38.50 (24.10, 97.25)	35.90 (23.60, 96.50)	50.00 (32.30, 106.90)	*Z* = 1.675	0.094
DB, umol/L, M (*Q*_1_, *Q*_3_)	3.65 (2.00, 9.95)	3.40 (2.00, 9.30)	4.20 (2.00, 15.80)	*Z* = 1.038	0.299
TB, umol/L, M (*Q*_1_, *Q*_3_)	18.65 (12.50, 29.45)	19.00 (13.00, 29.40)	17.70 (9.90, 32.20)	*Z* = −0.200	0.841
GGT, U/L, M (*Q*_1_, *Q*_3_)	106.75 (40.80, 292.85)	107.80 (41.00, 319.00)	97.00 (32.20, 255.70)	*Z* = −0.928	0.354
AKP, U/L, M (*Q*_1_, *Q*_3_)	86.25 (63.75, 142.50)	87.60 (65.00, 146.00)	66.50 (57.30, 110.80)	*Z* = −1.938	0.053
AMY, U/L, M (*Q*_1_, *Q*_3_)	223.50 (86.00, 619.00)	198.00 (86.00, 534.00)	449.00 (102.00, 1049.00)	*Z* = 2.146	0.032
LPS, U/L, M (*Q*_1_, *Q*_3_)	364.00 (96.00, 1073.00)	373.00 (92.00, 1171.00)	362.00 (98.00, 787.00)	*Z* = −0.544	0.586
BUN, mmol/L, M (*Q*_1_, *Q*_3_)	5.00 (3.69, 6.95)	4.86 (3.65, 6.60)	6.60 (5.00, 9.30)	*Z* = 3.721	<0.001
Scr, umol/L, M (*Q*_1_, *Q*_3_)	68.70 (56.00, 86.90)	68.00 (55.60, 81.90)	84.40 (63.50, 149.00)	*Z* = 2.993	0.003
Ca, mmol/L, Mean ± SD	2.06 ± 0.25	2.11 ± 0.19	1.75 ± 0.35	*t* = 5.93	<0.001
LDH, U/L, M (*Q*_1_, *Q*_3_)	309.00 (205.50, 527.00)	287.00 (200.00, 443.00)	655.00 (492.00, 819.00)	*Z* = 6.181	<0.001
Day 1 admission					
CRP level, mg/L, Mean ± SD	83.92 ± 94.29	71.21 ± 78.15	174.31 ± 140.96	*t* = −4.24	<0.001
ALB level, g/L, Mean ± SD	38.37 ± 6.64	39.19 ± 6.39	32.53 ± 5.34	*t* = 5.88	<0.001
CRP/ALB, Mean ± SD	2.40 ± 2.84	1.95 ± 2.18	5.63 ± 4.48	*t* = −4.79	<0.001
Day 2 admission					
CRP level, mg/L, Mean ± SD	125.74 ± 110.07	104.42 ± 87.64	277.43 ± 133.43	*t* = −7.45	<0.001
ALB level, g/L, Mean ± SD	37.14 ± 6.09	37.81 ± 5.51	32.34 ± 7.75	*t* = 4.03	<0.001
CRP/ALB, M (*Q*_1_, *Q*_3_)	2.85 (0.84,5.36)	2.22 (0.75,4.73)	7.46 (5.90,12.00)	*Z* = 7.561	<0.001
Day 3 admission					
CRP level, mg/L, Mean ± SD	81.12 ± 85.80	63.71 ± 62.68	204.93 ± 120.90	*t* = −6.78	<0.001
ALB level, g/L, Mean ± SD	36.82 ± 6.00	37.71 ± 5.51	30.49 ± 5.59	*t* = 7.25	<0.001
CRP/ALB, Mean ± SD	2.47 ± 2.90	1.83 ± 1.95	7.03 ± 4.27	*t* = −7.09	<0.001

Notes: SAP, severe acute pancreatitis; MCTSI, modified computed tomography severity index; BISAP, Bedside Index of Severity in Acute Pancreatitis; WBC, white blood cell; Hb, hemoglobin; hemoglobin; PLR, platelet to lymphocyte ratio; NLR, neutrophil to lymphocyte ratio; ALT, alanine transaminase; AST, aspartate aminotransferase; DB, direct bilirubin; TB, total bilirubin; GGT, gamma-glutamyl transpeptidase; AKP, alkaline phosphatase; AMY, amylase; LPS, lipase; BUN, blood urea nitrogen; Scr, serum creatinine; Ca, calcium; LDH, lactate dehydrogenase; CRP, C-reactive protein; ALB, serum albumin.

Thirty-five patients had SAP. There were statistically significant differences in hyperlipidemia, WBC, neutrophil counts, PLR, NLR, AMY, BUN, Scr, Ca, and LDH between patients with and without SAP, which may be the factors affecting SAP. Differences in population characteristics between patients with and without SAP are shown in Table 1.

Finally, 11 patients died. Ranson, MCTSI, BISAP, WBC, neutrophil counts, PLR, NLR, AMY, BUN, Scr, Ca, LDH, CRP, ALB, CRP/ALB on day 2, and CRP, ALB, CRP/ALB on day 3 may be associated with the death of AP. Differences in population characteristics between death and survival are depicted in Table 2.

**Table 2:** Differences of population characteristics between death and survival of AP patients.

Variables	Total (*n* = 284)	Survival (*n* = 273)	Death (*n* = 11)	Statistics	*P*
Gender, *n* (%)				–	1.000
Female	123 (43.31)	118 (43.22)	5 (45.45)		
Male	161 (56.69)	155 (56.78)	6 (54.55)		
Age, years, Mean ± SD	57.26 ± 18.12	57.33 ± 18.18	55.55 ± 17.10	*t* = 0.32	0.749
**Etiology**					
Gallstone, *n* (%)				*χ*^2^ = 0.408	0.523
No	130 (45.77)	126 (46.15)	4 (36.36)		
Yes	154 (54.23)	147 (53.85)	7 (63.64)		
Hyperlipidemia, *n* (%)				–	0.143
No	215 (75.70)	209 (76.56)	6 (54.55)		
Yes	69 (24.30)	64 (23.44)	5 (45.45)		
Alcohol, *n* (%)				–	0.084
No	271 (95.42)	262 (95.97)	9 (81.82)		
Yes	13 (4.58)	11 (4.03)	2 (18.18)		
Other, *n* (%)				*χ*^2^ = 3.130	0.077
No	223 (78.52)	212 (77.66)	11 (100.00)		
Yes	61 (21.48)	61 (22.34)	0 (0.00)		
Ranson, M (*Q*_1_, *Q*_3_)	2.00 (1.00, 3.00)	2.00 (1.00, 3.00)	6.00 (4.00, 7.00)	*Z* = 4.851	<0.001
MCTSI, M (*Q*_1_, *Q*_3_)	2.00 (2.00, 4.00)	2.00 (2.00, 4.00)	6.00 (6.00, 8.00)	*Z* = 4.335	<0.001
BISAP, M (*Q*_1_, *Q*_3_)	1.00 (0.00, 2.00)	1.00 (0.00, 2.00)	3.00 (3.00, 4.00)	*Z* = 5.241	<0.001
WBC, 10^9^/L, M (*Q*_1_, *Q*_3_)	10.48 (7.44, 14.46)	10.37 (7.32, 14.10)	21.31 (9.85, 24.71)	*Z* = 3.084	0.002
Hb, g/L, Mean ± SD	130.70 ± 21.10	130.40 ± 20.24	138.36 ± 37.13	*t* = −0.71	0.495
Neutrophil counts, 10^9^/L, M (*Q*_1_, *Q*_3_)	8.56 (5.80, 12.26)	8.44 (5.71, 11.92)	19.03 (8.03,23.01)	*Z* = 3.203	0.001
Lymphocyte counts, 10^9^/L, M (*Q*_1_, *Q*_3_)	1.15 (0.82,1.48)	1.17 (0.82,1.50)	1.00 (0.74,1.13)	*Z* = −1.341	0.180
Platelet counts, 10^9^/L, M (*Q*_1_, *Q*_3_)	196.00 (150.50,245.00)	194.00 (150.00,241.00)	259.00 (191.00,273.00)	*Z* = 1.957	0.050
PLR, M (*Q*_1_, *Q*_3_)	178.05 (119.44,242.36)	175.94 (118.97,236.67)	253.33 (169.03,440.32)	*Z* = 2.239	0.025
NLR, M (*Q*_1_, *Q*_3_)	7.84 (4.67,13.31)	7.66 (4.61,11.94)	14.82 (12.50,29.65)	*Z* = 3.329	<0.001
ALT, U/L, M (*Q*_1_, *Q*_3_)	42.70 (24.55, 145.20)	43.40 (24.70, 144.10)	37.90 (22.50, 161.50)	*Z* = −0.043	0.966
AST, U/L, M (*Q*_1_, *Q*_3_)	38.50 (24.10, 97.25)	37.70 (23.80, 96.50)	65.60 (38.60, 106.90)	*Z* = 1.724	0.085
DB, umol/L, M (*Q*_1_, *Q*_3_)	3.65 (2.00, 9.95)	3.60 (2.00, 9.70)	4.00 (2.00, 14.20)	*Z* = −0.017	0.986
TB, umol/L, M (*Q*_1_, *Q*_3_)	18.65 (12.50, 29.45)	19.40 (12.60, 29.40)	17.40 (11.30, 30.40)	*Z* = −0.399	0.690
GGT, M (*Q*_1_, *Q*_3_)	106.75 (40.80, 292.85)	105.70 (40.70, 289.70)	117.40 (48.10, 304.20)	*Z* = 0.455	0.649
AKP, U/L, M (*Q*_1_, *Q*_3_)	86.25 (63.75, 142.50)	86.40 (64.20, 145.80)	66.50 (61.90, 109.00)	*Z* = −0.826	0.409
AMY, U/L, M (*Q*_1_, *Q*_3_)	223.50 (86.00, 619.00)	210.00 (85.00, 571.00)	1190.00 (249.00, 1200.00)	*Z* = 2.835	0.005
LPS, U/L, M (*Q*_1_, *Q*_3_)	364.00 (96.00, 1073.00)	357.00 (95.00, 1073.00)	787.00 (230.00, 1201.00)	*Z* = 0.646	0.518
BUN, mmol/L, M (*Q*_1_, *Q*_3_)	5.00 (3.69, 6.95)	4.95 (3.68, 6.80)	7.30 (5.40, 11.30)	*Z* = 2.762	0.006
Scr, umol/L, M (*Q*_1_, *Q*_3_)	68.70 (56.00, 86.90)	68.00 (55.70, 83.80)	95.10 (72.00, 150.90)	*Z* = 3.033	0.002
Ca, mmol/L, Mean ± SD	2.06 ± 0.25	2.08 ± 0.23	1.72 ± 0.35	*t* = 3.39	0.007
LDH, U/L, M (*Q*_1_, *Q*_3_)	309.00 (205.50, 527.00)	307.00 (205.00, 504.00)	655.00 (302.00, 953.00)	*Z* = 2.675	0.007
Day 1 admission					
CRP level, mg/L, Mean ± SD	83.92 ± 94.29	80.26 ± 88.41	174.72 ± 171.95	*t* = −1.81	0.099
ALB level, g/L, Mean ± SD	38.37 ± 6.64	38.52 ± 6.60	34.65 ± 6.96	*t* = 1.90	0.058
CRP/ALB, Mean ± SD	2.40 ± 2.84	2.28 ± 2.64	5.48 ± 5.24	*t* = −2.02	0.071
Day 2 admission					
CRP level, mg/L, Mean ± SD	125.74 ± 110.07	118.24 ± 101.22	311.77 ± 156.93	*t* = −4.06	0.002
ALB level, g/L, Mean ± SD	37.14 ± 6.09	37.50 ± 5.87	27.98 ± 3.91	*t* = 5.32	<0.001
CRP/ALB, M (*Q*_1_, *Q*_3_)	2.85 (0.84, 5.36)	2.68 (0.82, 5.19)	12.00 (7.26, 14.42)	*Z* = 4.254	<0.001
Day 3 admission					
CRP level, mg/L, Mean ± SD	81.12 ± 85.80	73.39 ± 74.06	273.02 ± 130.38	*t* = −5.05	<0.001
ALB level, g/L, Mean ± SD	36.82 ± 6.00	37.11 ± 5.86	29.69 ± 5.13	*t* = 4.13	<0.001
CRP/ALB, Mean ± SD	2.47 ± 2.90	2.20 ± 2.49	9.24 ± 4.14	*t* = −5.59	<0.001
Hospitalization length, Days, M (*Q*_1_, *Q*_3_)	9.00 (8.00, 13.00)	9.00 (8.00, 13.00)	17.00 (7.00, 26.00)	*Z* = 0.793	0.428

Notes: AP, acute pancreatitis; other etiologies include, autoimmune diseases, metabolic diseases, trauma or postoperative, drugs, viral infection, etc., or the specific etiologies of pancreatitis cannot be determined; MCTSI, modified computed tomography severity index; BISAP, Bedside Index of Severity in Acute Pancreatitis; WBC, white blood cell; Hb, hemoglobin; hemoglobin; PLR, platelet to lymphocyte ratio; NLR, neutrophil to lymphocyte ratio; ALT, alanine transaminase; AST, aspartate aminotransferase; DB, direct bilirubin; TB, total bilirubin; GGT, gamma-glutamyl transpeptidase; AKP, alkaline phosphatase; AMY, amylase; LPS, lipase; BUN, blood urea nitrogen; Scr, serum creatinine; Ca, calcium; LDH, lactate dehydrogenase; CRP, C-reactive protein; ALB, serum albumin.

Pancreatic necrosis occurred in 29 patients. The AP patients with pancreatic necrosis were younger than patients without pancreatic necrosis. Hyperlipidemia may be the factor affecting pancreatic necrosis in AP patients. Higher levels of Ranson, MCTSI, BISAP, WBC, neutrophil counts, NLR, BUN, Ca, LDH, CRP, CRP/ALB were observed in AP patients with pancreatic necrosis. The differences between patients with and without pancreatic necrosis are shown in Table 3.

**Table 3:** Differences of population characteristics between patients with and without pancreatic necrosis.

Variables	Total (*n* = 284)	Non-pancreatic necrosis (*n* = 255)	Pancreatic necrosis (*n* = 29)	Statistics	*P*
Gender, *n* (%)				*χ*^2^ = 0.049	0.825
Female	123 (43.31)	111 (43.53)	12 (41.38)		
Male	161 (56.69)	144 (56.47)	17 (58.62)		
Age, years, M (*Q*_1_, *Q*_3_)	59.50 (39.00, 70.00)	61.00 (41.00, 70.00)	41.00 (32.00, 57.00)	*Z* = −3.438	<0.001
**Etiology**					
Gallstone, *n* (%)				*χ*^2^ = 3.455	0.063
No	130 (45.77)	112 (43.92)	18 (62.07)		
Yes	154 (54.23)	143 (56.08)	11 (37.93)		
Hyperlipidemia, *n* (%)				*χ*^2^ = 7.403	0.007
No	215 (75.70)	199 (78.04)	16 (55.17)		
Yes	69 (24.30)	56 (21.96)	13 (44.83)		
Alcohol, *n* (%)				–	0.136
No	271 (95.42)	245 (96.08)	26 (89.66)		
Yes	13 (4.58)	10 (3.92)	3 (10.34)		
Other, *n* (%)				*χ*^2^ = 0.012	0.913
No	223 (78.52)	200 (78.43)	23 (79.31)		
Yes	61 (21.48)	55 (21.57)	6 (20.69)		
Ranson, M (*Q*_1_, *Q*_3_)	2.00 (1.00, 3.00)	2.00 (1.00, 3.00)	4.00 (3.00, 5.00)	*Z* = 5.363	<0.001
MCTSI, M (*Q*_1_, *Q*_3_)	2.00 (2.00, 4.00)	2.00 (2.00, 4.00)	6.00 (6.00, 8.00)	*Z* = 7.191	<0.001
BISAP, M (*Q*_1_, *Q*_3_)	1.00 (0.00, 2.00)	1.00 (0.00, 2.00)	2.00 (1.00, 3.00)	*Z* = 4.420	<0.001
WBC, M (*Q*_1_, *Q*_3_)	10.48 (7.44, 14.46)	10.21 (7.30,13.53)	14.50 (9.43,18.24)	*Z* = 3.085	0.002
Hb, g/L, Mean ± SD	130.70 ± 21.10	130.49 ± 20.08	132.62 ± 28.89	*t* = −0.39	0.701
Neutrophil counts, 10^9^/L, M (*Q*_1_, *Q*_3_)	8.56 (5.80,12.26)	8.35 (5.64,11.84)	12.24 (8.14,15.39)	*Z* = 3.260	0.001
Lymphocyte counts, 10^9^/L, M (*Q*_1_, *Q*_3_)	1.15 (0.82, 1.48)	1.17 (0.82, 1.50)	1.06 (0.83, 1.37)	*Z* = −0.375	0.708
Platelet counts, 10^9^/L, M (*Q*_1_, *Q*_3_)	196.00 (150.50, 245.00)	195.00 (151.00, 245.00)	199.00 (146.00, 254.00)	*Z* = −0.072	0.943
PLR, M (*Q*_1_, *Q*_3_)	178.05 (119.44, 242.36)	177.88 (119.82, 242.50)	180.31 (117.07, 229.92)	*Z* = −0.555	0.579
NLR, M (*Q*_1_, *Q*_3_)	7.84 (4.67, 13.31)	7.04 (4.54, 13.23)	10.95 (7.79, 13.50)	*Z* = 2.923	0.003
ALT, U/L, M (*Q*_1_, *Q*_3_)	42.70 (24.55, 145.20)	43.80 (24.70, 151.70)	32.50 (22.80, 54.90)	*Z* = −1.423	0.155
AST, U/L, M (*Q*_1_, *Q*_3_)	38.50 (24.10, 97.25)	37.70 (23.40, 103.60)	40.00 (30.90, 70.30)	*Z* = 0.827	0.408
DB, umol/L, M (*Q*_1_, *Q*_3_)	3.65 (2.00, 9.95)	3.50 (2.00, 9.90)	4.20 (2.00, 11.00)	*Z* = 0.703	0.482
TB, umol/L, M (*Q*_1_, *Q*_3_)	18.65 (12.50, 29.45)	18.30 (12.40, 28.70)	19.80 (13.60, 32.20)	*Z* = 0.511	0.610
GGT, M (*Q*_1_, *Q*_3_)	106.75 (40.80, 292.85)	108.00 (39.20, 319.00)	101.20 (48.10, 148.70)	*Z* = −1.098	0.272
AKP, U/L, M (*Q*_1_, *Q*_3_)	86.25 (63.75, 142.50)	87.10 (64.20, 154.00)	74.50 (59.40, 107.80)	*Z* = −1.410	0.158
AMY, U/L, M (*Q*_1_, *Q*_3_)	223.50 (86.00, 619.00)	220.00 (86.00, 618.00)	249.00 (102.00, 647.00)	*Z* = 0.635	0.526
LPS, U/L, M (*Q*_1_, *Q*_3_)	364.00 (96.00, 1073.00)	381.00 (92.00, 1146.00)	338.00 (107.00, 574.00)	*Z* = −1.000	0.317
BUN, mmol/L, M (*Q*_1_, *Q*_3_)	5.00 (3.69, 6.95)	4.90 (3.66, 6.70)	5.96 (4.40, 9.00)	*Z* = 2.191	0.028
Scr, umol/L, M (*Q*_1_, *Q*_3_)	68.70 (56.00, 86.90)	68.10 (55.40, 86.00)	70.70 (63.50, 97.00)	*Z* = 1.901	0.057
Ca, mmol/L, Mean ± SD	2.06 ± 0.25	2.09 ± 0.22	1.84 ± 0.35	*t* = 3.71	<0.001
LDH, U/L, M (*Q*_1_, *Q*_3_)	309.00 (205.50, 527.00)	300.00 (204.00, 492.00)	504.00 (377.00, 764.00)	*Z* = 3.385	<0.001
Day 1 admission					
CRP level, mg/L, Mean ± SD	83.92 ± 94.29	74.89 ± 84.21	163.32 ± 135.14	*t* = −3.45	0.002
ALB level, g/L, Mean ± SD	38.37 ± 6.64	38.82 ± 6.53	34.39 ± 6.33	*t* = 3.47	<0.001
CRP/ALB, MEAN ± SD	2.40 ± 2.84	2.09 ± 2.43	5.16 ± 4.37	*t* = −3.73	<0.001
Day 2 admission					
CRP level, mg/L, Mean ± SD	125.74 ± 110.07	113.10 ± 100.69	236.89 ± 127.41	*t* = −6.09	<0.001
ALB level, g/L, Mean ± SD	37.14 ± 6.09	37.51 ± 5.86	33.87 ± 7.12	*t* = 3.10	0.002
CRP/ALB, M (*Q*_1_, *Q*_3_)	2.85 (0.84, 5.36)	2.39 (0.78, 5.00)	5.98 (4.71, 8.41)	*Z* = 5.140	<0.001
Day 3 admission					
CRP level, mg/L, Mean ± SD	81.12 ± 85.80	72.11 ± 77.34	160.31 ± 113.58	*t* = −4.08	<0.001
ALB level, g/L, Mean ± SD	36.82 ± 6.00	37.32 ± 5.89	32.47 ± 5.18	*t* = 4.24	<0.001
CRP/ALB, Mean ± SD	2.47 ± 2.90	2.16 ± 2.59	5.24 ± 3.93	*t* = −4.12	<0.001

Notes: MCTSI, modified computed tomography severity index; BISAP, Bedside Index of Severity in Acute Pancreatitis; WBC, white blood cell; Hb, hemoglobin; hemoglobin; PLR, platelet to lymphocyte ratio; NLR, neutrophil to lymphocyte ratio; ALT, alanine transaminase; AST, aspartate aminotransferase; DB, direct bilirubin; TB, total bilirubin; GGT, gamma-glutamyl transpeptidase; AKP, alkaline phosphatase; AMY, amylase; LPS, lipase; BUN, blood urea nitrogen; Scr, serum creatinine; Ca, calcium; LDH, lactate dehydrogenase; CRP, C-reactive protein; ALB, serum albumin.

Table 4 shows the difference in population characteristics between organ failure and without organ failure. There were statistically significant differences in WBC, NLR, AST, DB, BUN, Scr, Ca and LDH between AP patients with organ failure, and those without organ failure.

**Table 4:** Differences of population characteristics between patients with and without organ failure.

Variables	Total (*n* = 284)	Non-organ failure (*n* = 255)	Organ failure (*n* = 29)	Statistics	*P*
Gender, *n* (%)				*χ*^2^ = 0.381	0.537
Female	123 (43.31)	112 (43.92)	11 (37.93)		
Male	161 (56.69)	143 (56.08)	18 (62.07)		
Age, years, M (*Q*_1_, *Q*_3_)	59.50 (39.00, 70.00)	59.00 (39.00, 70.00)	61.00 (39.00, 76.00)	*Z* = 0.592	0.554
**Etiology**					
Gallstone, *n* (%)				*χ*^2^ = 0.251	0.616
No	130 (45.77)	118 (46.27)	12 (41.38)		
Yes	154 (54.23)	137 (53.73)	17 (58.62)		
Hyperlipidemia, *n* (%)				*χ*^2^ = 0.797	0.372
No	215 (75.70)	195 (76.47)	20 (68.97)		
Yes	69 (24.30)	60 (23.53)	9 (31.03)		
Alcohol, *n* (%)				–	0.630
No	271 (95.42)	244 (95.69)	27 (93.10)		
Yes	13 (4.58)	11 (4.31)	2 (6.90)		
Other, *n* (%)				*χ*^2^ = 2.374	0.123
No	223 (78.52)	197 (77.25)	26 (89.66)		
Yes	61 (21.48)	58 (22.75)	3 (10.34)		
Ranson, M (*Q*_1_, *Q*_3_)	2.00 (1.00, 3.00)	1.00 (1.00, 3.00)	5.00 (4.00, 6.00)	*Z* = 8.152	<0.001
MCTSI, M (*Q*_1_, *Q*_3_)	2.00 (2.00, 4.00)	2.00 (2.00, 4.00)	6.00 (4.00, 8.00)	*Z* = 6.273	<0.001
BISAP, M (*Q*_1_, *Q*_3_)	1.00 (0.00, 2.00)	1.00 (0.00, 1.00)	3.00 (2.00, 3.00)	*Z* = 8.168	<0.001
WBC, M (*Q*_1_, *Q*_3_)	10.48 (7.44, 14.46)	10.32 (7.30, 13.72)	14.94 (9.81, 21.35)	*Z* = 3.044	0.002
Hb, g/L, Mean ± SD	130.70 ± 21.10	131.33 ± 19.70	125.21 ± 30.68	*t* = 1.05	0.302
Neutrophil counts, 10^9^/L, M (*Q*_1_, *Q*_3_)	8.56 (5.80, 12.26)	8.35 (5.58, 11.84)	13.23 (8.03, 19.03)	*Z* = 3.262	0.001
Lymphocyte counts, 10^9^/L, M (*Q*_1_, *Q*_3_)	1.15 (0.82, 1.48)	1.17 (0.83, 1.50)	1.04 (0.74, 1.38)	*Z* = −1.311	0.190
Platelet counts, 10^9^/L, M (*Q*_1_, *Q*_3_)	196.00 (150.50, 245.00)	195.00 (151.00, 241.00)	212.00 (137.00, 270.00)	*Z* = 0.426	0.670
PLR, M (*Q*_1_, *Q*_3_)	178.05 (119.44, 242.36)	175.00 (118.42, 239.39)	197.73 (147.31, 259.00)	*Z* = 1.358	0.175
NLR, M (*Q*_1_, *Q*_3_)	7.84 (4.67, 13.31)	7.53 (4.54, 11.83)	12.50 (7.44, 21.28)	*Z* = 3.516	<0.001
ALT, U/L, M (*Q*_1_, *Q*_3_)	42.70 (24.55, 145.20)	42.00 (24.60, 149.00)	46.20 (24.50, 127.30)	*Z* = 0.424	0.672
AST, U/L, M (*Q*_1_, *Q*_3_)	38.50 (24.10, 97.25)	35.90 (22.80, 94.90)	73.20 (38.60, 110.70)	*Z* = 2.497	0.013
DB, umol/L, M (*Q*_1_, *Q*_3_)	3.65 (2.00, 9.95)	3.30 (2.00, 9.30)	5.90 (2.50, 17.80)	*Z* = 2.006	0.045
TB, umol/L, M (*Q*_1_, *Q*_3_)	18.65 (12.50, 29.45)	19.40 (13.00, 29.10)	17.40 (11.00, 47.80)	*Z* = −0.255	0.798
GGT, M (*Q*_1_, *Q*_3_)	106.75 (40.80, 292.85)	104.30 (39.20, 314.60)	128.60 (63.00, 256.10)	*Z* = 0.537	0.591
AKP, U/L, M (*Q*_1_, *Q*_3_)	86.25 (63.75, 142.50)	86.20 (64.20, 137.00)	86.40 (62.20, 171.40)	*Z* = −0.141	0.888
AMY, U/L, M (*Q*_1_, *Q*_3_)	223.50 (86.00, 619.00)	210.00 (85.00, 541.00)	399.00 (125.00, 1049.00)	*Z* = 1.960	0.050
LPS, U/L, M (*Q*_1_, *Q*_3_)	364.00 (96.00, 1073.00)	357.00 (92.00, 1073.00)	421.00 (98.00, 1027.00)	*Z* = 0.227	0.821
BUN, mmol/L, M (*Q*_1_, *Q*_3_)	5.00 (3.69, 6.95)	4.90 (3.60, 6.60)	8.20 (5.40, 12.60)	*Z* = 4.736	<0.001
Scr, umol/L, M (*Q*_1_, *Q*_3_)	68.70 (56.00, 86.90)	67.80 (55.00, 81.60)	97.00 (72.00, 155.40)	*Z* = 4.833	<0.001
Ca, mmol/L, Mean ± SD	2.06 ± 0.25	2.09 ± 0.22	1.82 ± 0.34	*t* = 4.25	<0.001
LDH, U/L, M (*Q*_1_, *Q*_3_)	309.00 (205.50, 527.00)	294.00 (200.00, 465.00)	658.00 (492.00, 911.00)	*Z* = 5.707	<0.001
Day 1 admission					
CRP level, mg/L, Mean ± SD	83.92 ± 94.29	76.23 ± 85.67	151.55 ± 134.21	*t* = −2.95	0.006
ALB level, g/L, Mean ± SD	38.37 ± 6.64	38.98 ± 6.48	33.00 ± 5.63	*t* = 4.76	<0.001
CRP/ALB, Mean ± SD	2.40 ± 2.84	2.11 ± 2.46	4.91 ± 4.38	*t* = −3.39	0.002
Day 2 admission					
CRP level, mg/L, Mean ± SD	125.74 ± 110.07	113.74 ± 97.14	231.21 ± 155.03	*t* = −3.99	<0.001
ALB level, g/L, Mean ± SD	37.14 ± 6.09	38.12 ± 5.42	28.47 ± 4.65	*t* = 9.21	<0.001
CRP/ALB, M (*Q*_1_, *Q*_3_)	2.85 (0.84, 5.36)	2.49 (0.80, 5.04)	7.16 (3.86, 13.37)	*Z* = 4.988	<0.001
Day 3 admission					
CRP level, mg/L, Mean ± SD	81.12 ± 85.80	70.71 ± 73.22	172.67 ± 126.87	*t* = −4.25	<0.001
ALB level, g/L, Mean ± SD	36.82 ± 6.00	37.54 ± 5.74	30.49 ± 4.33	*t* = 6.41	<0.001
CRP/ALB, Mean ± SD	2.47 ± 2.90	2.10 ± 2.45	5.77 ± 4.26	*t* = −4.55	<0.001

Notes: MCTSI, modified computed tomography severity index; BISAP, Bedside Index of Severity in Acute Pancreatitis; WBC, white blood cell; Hb, hemoglobin; hemoglobin; PLR, platelet to lymphocyte ratio; NLR, neutrophil to lymphocyte ratio; ALT, alanine transaminase; AST, aspartate aminotransferase; DB, direct bilirubin; TB, total bilirubin; GGT, gamma-glutamyl transpeptidase; AKP, alkaline phosphatase; AMY, amylase; LPS, lipase; BUN, blood urea nitrogen; Scr, serum creatinine; Ca, calcium; LDH, lactate dehydrogenase; CRP, C-reactive protein; ALB, serum albumin.

### Evaluations of the prognosis values of CRP/ALB ratio, Ranson, MCTSI, and BISAP in AP

CRP/ALB ratio on day 1 was related to SAP [odds ratio (OR): 1.32, 95% confidence interval (CI): 1.12 to 1.55, *P *< 0.001), pancreatic necrosis (OR: 1.20, 95% CI: 1.04 to 1.38, *P *= 0.010), however, CRP/ALB ratio on day 1 was not related to death (OR: 1.22, 95% CI: 0.98 to 1.53, *P *= 0.078), and organ failure (OR: 1.09, 95% CI: 0.95 to 1.26, *P *= 0.220). CRP/ALB ratio on day 2 was associated with SAP (OR: 1.74, 95% CI: 1.32 to 2.29, *P *< 0.001), death (OR: 1.73, 95%CI: 1.24 to 2.41, *P *= 0.001), pancreatic necrosis (OR: 1.28, 95%CI: 1.08 to 1.50, *P *= 0.003), and organ failure (OR: 1.43, 95%CI: 1.18 to 1.73, *P *< 0.001) in AP patients. Similarly, CRP/ALB on day 3 was related to higher risk of SAP (OR: 1.50, 95%CI: 1.24 to 1.81, *P *< 0.001), death (OR: 1.8, 95%CI: 1.34 to 2.65, *P *< 0.001), pancreatic necrosis (OR: 1.22, 95%CI: 1.04 to 1.42, *P *= 0.014), and organ failure (OR: 1.21, 95%CI: 1.04 to 1.41, *P *= 0.015). Ranson, MCTSI, and BISAP scores could also predict the risk of SAP, death, pancreatic necrosis, and organ failure in AP patients. Evaluations of the prognosis values of CRP /ALB, Ranson, MCTSI, and BISAP in AP patients are presented in Table 5.

**Table 5:** The prognosis values of CRP/ALB ratio, ranson, MCTSI, and BISAP in AP.

Outcomes	Variables	Model 1		Model 2		Model 3	
		OR (95%CI)	*P*	OR (95%CI)	*P*	OR (95%CI)	*P*
SAP							
	CRP/ALB						
	Day1	1.44 (1.27–1.63)	<0.001	1.44 (1.27–1.62)	<0.001	1.32 (1.12–1.55)	<0.001
	Day2	1.79 (1.48–2.17)	<0.001	1.81 (1.49–2.19)	<0.001	1.74 (1.32–2.29)	<0.001
	Day3	1.77 (1.49–2.09)	<0.001	1.77 (1.49–2.09)	<0.001	1.50 (1.24–1.81)	<0.001
	Ranson	3.36 (2.35–4.81)	<0.001	3.44 (2.38–4.96)	<0.001	2.96 (1.80–4.87)	<0.001
	MCTSI	2.61 (1.96–3.48)	<0.001	2.74 (2.01–3.74)	<0.001	2.33 (1.62–3.35)	<0.001
	BISAP	7.83 (4.30–14.26)	<0.001	12.14 (5.85–25.21)	<0.001	12.21 (4.2734.94)	<0.001
Death							
	CRP/ALB						
	Day1	1.29 (1.11–1.50)	0.001	1.30 (1.11–1.52)	<0.001	1.22 (0.98–1.53)	0.078
	Day2	1.49 (1.27–1.74)	<0.001	1.53 (1.29–1.80)	<0.001	1.73 (1.24–2.41)	0.001
	Day3	1.57 (1.32–1.88)	<0.001	1.62 (1.34–1.96)	<0.001	1.88 (1.34–2.65)	<0.001
	Ranson	2.90 (1.84–4.55)	<0.001	3.14 (1.87–5.27)	<0.001	9.66 (1.90–49.21)	0.006
	MCTSI	2.03 (1.45–2.83)	<0.001	2.13 (1.47–3.08)	<0.001	1.86 (1.18–2.93)	0.008
	BISAP	6.72 (3.04–14.86)	<0.001	7.19 (3.07–16.86)	<0.001	7.07 (2.13–23.51)	0.001
Pancreatic necrosis							
	CRP/ALB						
	Day1	1.33 (1.18–1.49)	<0.001	1.33 (1.18–1.50)	<0.001	1.20 (1.04–1.38)	0.010
	Day2	1.32 (1.19–1.47)	<0.001	1.34 (1.19–1.51)	<0.001	1.28 (1.08–1.50)	0.003
	Day3	1.29 (1.16–1.44)	<0.001	1.31 (1.17–1.47)	<0.001	1.22 (1.04–1.42)	0.014
	Ranson	1.82 (1.46–2.27)	<0.001	1.91 (1.52–2.39)	<0.001	1.94 (1.31–2.88)	<0.001
	MCTSI	2.55 (1.89–3.43)	<0.001	2.47 (1.82–3.36)	<0.001	2.49 (1.74–3.58)	<0.001
	BISAP	2.53 (1.74–3.68)	<0.001	3.54 (2.32–5.40)	<0.001	3.00 (1.62–5.58)	<0.001
Organ failure							
	CRP/ALB						
	Day1	1.30 (1.16–1.45)	<0.001	1.30 (1.16–1.47)	<0.001	1.09 (0.95–1.26)	0.220
	Day2	1.46 (1.28–1.67)	<0.001	1.48 (1.29–1.69)	<0.001	1.43 (1.18–1.73)	<0.001
	Day3	1.36 (1.21–1.53)	<0.001	1.37 (1.21–1.54)	<0.001	1.21 (1.04–1.41)	0.015
	Ranson	4.56 (2.76–7.52)	<0.001	4.67 (2.79–7.82)	<0.001	7.66 (3.26–17.99)	<0.001
	MCTSI	1.89 (1.52–2.35)	<0.001	2.09 (1.62–2.68)	<0.001	1.76 (1.31–2.36)	<0.001
	BISAP	11.37 (5.35–24.13)	<0.001	12.06 (5.56–26.16)	<0.001	10.70 (4.21–27.22)	<0.001

Notes: MCTSI, modified computed tomography severity index; BISAP, Bedside Index of Severity in Acute Pancreatitis; CRP, C-reactive protein; ALB, serum albumin; AP, acute pancreatitis; SAP, severe acute pancreatitis; OR, odds ratio; CI, confidence interval; Model 1 was an unadjusted model; Model 2 adjusted for gender and age; Model 3 for SAP adjusted for hyperlipidemia, WBC, neutrophil counts, PLR, NLR, AMY, BUN, Scr, Ca, LDH, CRP, and ALB; Model 3 for death adjusted for WBC, neutrophil counts, PLR, NLR, AMY, BUN, Scr, Ca, LDH, CRP, and ALB; Model 3 for pancreatic necrosis adjusted for hyperlipidemia, WBC, neutrophil counts, NLR, BUN, Ca, LDH, CRP, and ALB; Model 3 for organ failure adjusted for WBC, NLR, AST, DB, BUN, Scr, Ca, LDH, CRP, and ALB.

### Comparison of predictive values between CRP/ALB ratio and Ranson, MCTSI, and BISAP

**Figure 1:**
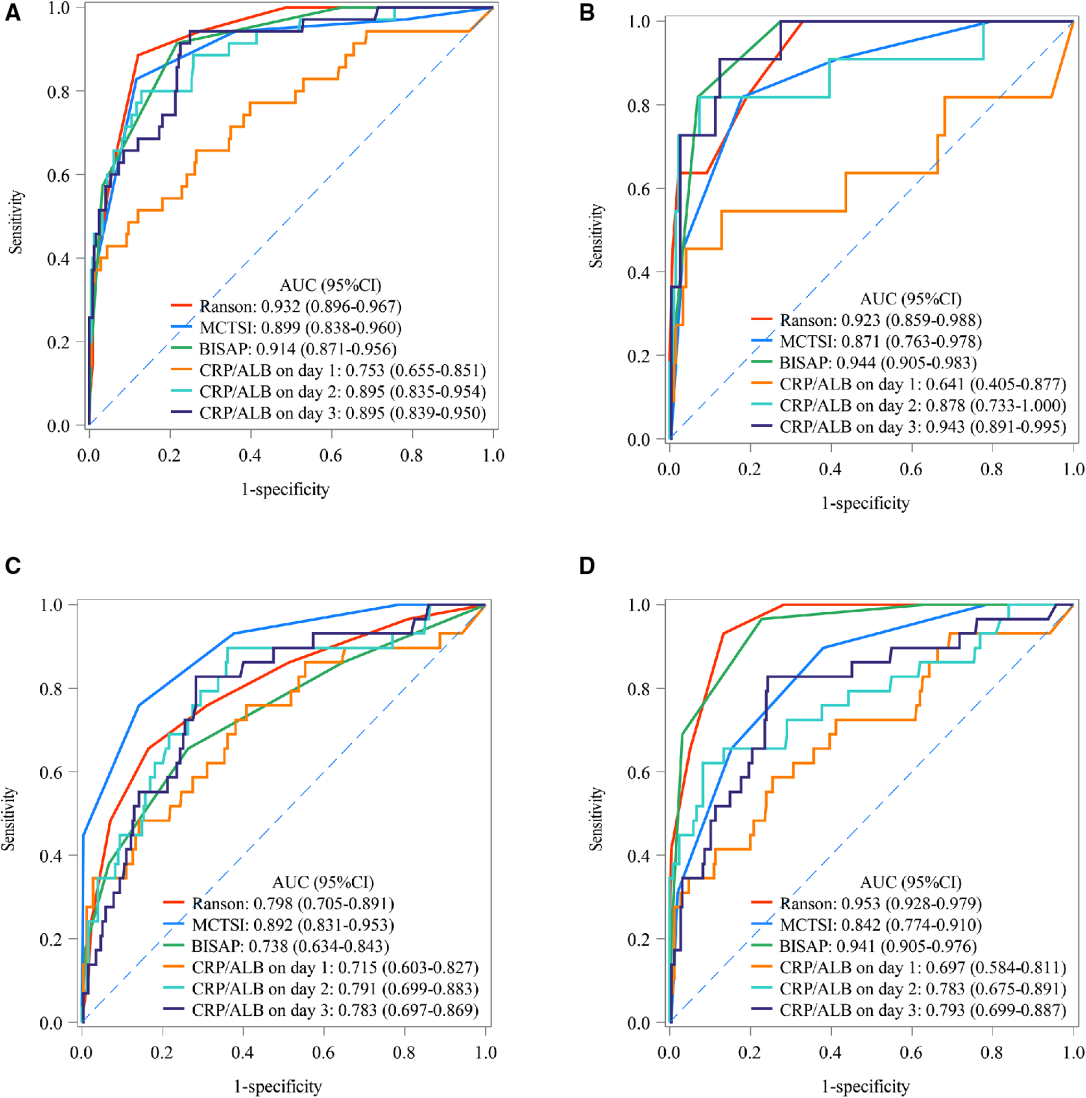
The AUC of CRP/ALB ratio, ranson, MCTSI, and BISAP in SAP, death, pancreatic necrosis, and organ failure; (**A**) SAP; (**B**) death; (**C**) pancreatic necrosis; (**D**) organ failure. MCTSI, modified computed tomography severity index; BISAP, bedside index of severity in acute pancreatitis; AUC, area under the curve; CRP, C-reactive protein; ALB, serum albumin; SAP, severe acute pancreatitis.

The predictive values of CRP/ALB ratio were compared with Ranson, MCTSI, and BISAP for predicting SAP, death, pancreatic necrosis, and organ failure among AP patients. The calculated AUC of the CRP/ALB ratio for SAP was 0.753 (95% CI: 0.655 to 0.852) on day 1, lowering than the AUC of Ranson (0.932, 95% CI: 0.896 to 0.967), MCTSI (0.899, 95% CI: 0.838 to 0.960), BISAP (0.914, 95% CI: 0.871 to 0.956), day 2 (0.895, 95% CI: 0.835 to 0.954), and day 3 (0.895, 95% CI: 0.839 to 0.950). The predictive value of CRP/ALB ratio on day 1 (AUC: 0.715, 95% CI: 0.603 to 0.828), day 2 (AUC: 0.791, 95% CI: 0.699 to 0.883), and day 3 (AUC: 0.783, 95% CI: 0.697 to 0.869) for pancreatic necrosis was lower than that of MCTSI (AUC: 0.892, 95% CI: 0.831 to 0.953). The predictive value of CRP/ALB ratio for organ failure on day 1 (AUC: 0.698, 95% CI: 0.675 to 0.891), day 2 (AUC: 0.783, 95% CI: 0.675 to 0.891), and day 3 (AUC: 0.793, 95% CI: 0.699 to 0.887) was lower than that of Ranson (AUC: 0.953, 95% CI: 0.928 to 0.979) and BISAP (AUC: 0.941, 95% CI: 0.905 to 0.976). The calculated AUC of the CRP/ALB ratio for death on day 3 was 0.943 (95% CI: 0.891 to 0.995), higher than the calculated AUC of the MCTSI (0.871, 95% CI: 0.763 to 0.978). Comparison outcomes of predictive values between CRP/ALB ratio and Ranson, MCTSI, and BISAP scores are shown in Table 6. Statistical power result indicated that the small sample size of CRP/ALB ratio on day 3 for death has not lowered statistical power. The predictive values of CRP/ALB ratio and Ranson, MCTSI, and BISAP are shown in Figure 1.

**Table 6:** Comparison results of predictive values between CRP/ALB ratio and ranson, MCTSI, BISAP.

Indicators	SAP				Pancreatic necrosis				Organ failure				Death			
	AUC (95%CI)	Sensitivity (95%CI)	Specificity (95%CI)	Cut-off	AUC (95%CI)	Sensitivity (95%CI)	Specificity (95%CI)	Cut-off	AUC (95%CI)	Sensitivity (95%CI)	Specificity (95%CI)	Cut-off	AUC (95%CI)	Sensitivity (95%CI)	Specificity (95%CI)	Cut-off
Ranson	0.932 (0.896–0.967)	0.571 (0.394–0.737)	0.952 (0.917–0.975)	4	0.798 (0.705–0.891)	0.483 (0.294–0.675)	0.929 (0.891–0.958)	4	0.953 (0.928–0.979)	0.931 (0.772–0.992)	0.867 (0.819–0.906	4	0.923 (0.859–0.988)	1.000 (0.715–1.000)	0.670 (0.611–0.726)	3
MCTSI	0.899 (0.838–0.960)	0.829 (0.664–0.934)	0.884 (0.837–0.921)	6	0.892 (0.831–0.953)	0.759 (0.565–0.897)	0.859 (0.810–0.899)	6	0.842 (0.774–0.910)	0.897 (0.726–0.978)	0.620 (0.557–0.679)	4	0.871 (0.763–0.978)	0.818 (0.482–0.977)	0.821 (0.770–0.864)	6
BISAP	0.914 (0.871–0.956)	1.000 (0.900–1.000)	0.378 (0.317–0.441)	1	0.738 (0.634–0.843)	0.655 (0.457–0.821)	0.737 (0.679–0.790)	2	0.941 (0.905–0.976)	0.690 (0.492–0.847)	0.969 (0.939–0.986)	2	0.944 (0.905–0.983)	0.818 (0.482–0.977)	0.930 (0.893–0.958)	3
**CRP/ALB**																
Day 1	0.753 (0.655–0.852)^a^,^b^,^c^	0.514 (0.340–0.686)^b^^,^^c^	0.880 (0.832–0.917)^a^^,^^c^	5.03	0.715 (0.603–0.828)^b^	0.724 (0.528–0.873)	0.592 (0.529–0.653)^a^^,^^b^,^c^	1.61	0.698 (0.675–0.891)^a^^,^^c^	0.586 (0.389–0.765)^a^^,^^b^	0.745 (0.687–0.797)^a^^,^^b^,^c^	3.12	0.641 (0.405–0.877)^a^^,^^c^	0.545 (0.234–0.833)^a^	0.872 (0.826–0.909)^a^	5.33
Day 2	0.895 (0.835–0.954)^d^	0.771 (0.599–0.896)^c^^,^^d^	0.871 (0.823–0.910)^a^^,^^c^,^d^	5.82	0.791 (0.699–0.883)^b^	0.897 (0.726–0.978)^a^	0.639 (0.577–0.698)^a^^,^^b^,^c^	3.86	0.783 (0.675–0.891)^a^^,^^c^	0.621 (0.423–0.793)^a^^,^^b^	0.918 (0.877–0.948)^b^^,^^c^,^d^	6.8	0.878 (0.733–1.000)	0.727 (0.390–0.940)	0.927 (0.889–0.955)^a^^,^^b^	7.26
Day 3	0.895 (0.839–0.950)^d^	0.943 (0.808–0.993)^a^^,^^d^	0.751 (0.692–0.803)^a^^,^^b^^,^^c^^,^^d^	2.37	0.783 (0.697–0.869)^b^	0.828 (0.642–0.942)^a^	0.718 (0.658–0.772)^a^^,^^b^,^d^	2.35	0.793 (0.699–0.887)^a^^,^^c^	0.793 (0.603–0.920)	0.757 (0.699–0.808)^a^^,^^b^^,^^c^^,^^d^	2.47	0.943 (0.891–0.995)^b^	0.818 (0.482–0.977)	0.875 (0.830–0.912)^a^	5.33

Notes: MCTSI, modified computed tomography severity index; BISAP, Bedside Index of Severity in Acute Pancreatitis; CRP, C-reactive protein; ALB, serum albumin; AP, acute pancreatitis; SAP, severe acute pancreatitis; CI, confidence interval; AUC, area under the curve.

^a^ Compared with Ranson, *P *< 0.05.

^b^ Compared with MCTSI, *P *< 0.05.

^c^ Compared with BISAP, *P *< 0.05.

^d^ Compared with Day 1 CRP /ALB ratio, *P *< 0.05.

^e^ Compared with CRP /ALB ratio, *P *< 0.05.

## Discussion

AP is a relatively common condition worldwide and is characterized by acute and severe upper abdominal pain (18). Accurate and timely identification of the severity and prognostic factors of AP patients is critical. Currently, Ranson Score, BISAP, and MCTSI were frequently used for early identification of the severity of AP (19). Each score has specific applications, but all have limitations. A simple, repeatable, and non-invasive laboratory procedure, is needed to predict the severity and prognostic factors of AP patients. In this study, we used the CRP/ALB ratio to predict SAP, death, pancreatic necrosis, and organ failure among AP patients. CRP/ALB ratio on day 1 was related to SAP and pancreatic necrosis, nevertheless, CRP/ALB ratio on day 1 was not related to death and organ failure. CRP/ALB ratio on day 2 and day 3 was associated with SAP, death, pancreatic necrosis, and organ failure in AP patients. The findings demonstrated the predictive value of the CRP/ALB ratio for the determination of the severity and prognosis of AP. However, CRP/ALB ratio may not superior to the Ranson, MCTSI, and BISAP scores. The predictive value of the CRP/ALB ratio may be helpful to the assessment of SAP and prognosis in AP patients.

CRP is a valuable marker of inflammation in the acute phase that is produced during infection, ischemia, and trauma and is synthesized by liver cells, smooth muscle cells, macrophages, endothelial cells, lymphocytes, and adipocytes in response to the regulation of pro-inflammatory cytokines, especially interleukin-6 (10, 20). Because of its short half-life and high sensitivity, CRP is often used for the detection and evaluation of inflammation (21). Serum ALB levels reflect patients' nutritional status; a low serum ALB level indicates a state of malnutrition (22). In previous studies, ALB has been shown to be inversely associated with inflammation severity, disease prognoses, and mortality in AP (23). The CRP/ALB ratio, a combined index of the ALB and CRP levels, is known to be related more consistently to prognosis than a single marker, accurately reflecting the degree of inflammation or nutritional deficiency (24–26). Many studies have investigated the prognostic value of the CRP/ALB ratio in a variety of diseases (14, 27, 28). The present study showed that day 2 and day 3 CRP/ALB ratio could predict the SAP and prognostic outcome of AP. A study by Wang et al. (29) reported that high levels of CRP and low ALB levels were associated with in-hospital mortality in patients with SAP. Zhao et al. found that the admission CRP/ALB ratio was significantly higher in the re-operation of AP patients under debridement (12). A study by Kaplan et al. indicated that the CRP/ALB ratio could predict the mortality of AP patients with a sensitivity of 92.1% and specificity of 58.0% (15). Nevertheless, in this study, the day 1 CRP/ALB ratio has not been confirmed to be useful to predict mortality and organ failure in AP patients. We speculate the possible reason for this result is the lower CRP/ALB ratio on the first day. When infection and inflammation occur, the level of serum CRP rises rapidly within a few hours, and reaches the peak in 24–48 h, however, serum ALB might be declined a few days after the initiation of pancreatic inflammation (30). Thus, combined CRP/ALB ratio could be increased along with progression of pancreatic inflammation due to aforementioned scenario. The cut-off value of the CRP/ALB ratio on the prognosis of AP needs further study. Moreover, the day 3 CRP/ALB ratio showed a better predictive performance in predicting mortality of AP patients compared with the day 2 CRP/ALB ratio. Similarly, Sun et al. found that CRP/ALB at 72 h may be one of the best indicators for the assessment of clinical therapy and prognosis of patients with sepsis (31). Monitoring of ambulatory CRP/ALB level changes in patients with AP may be necessary. All in all, CRP/ALB ratio may be an easy-to-measure, reproducible, non-invasive tool to predict the SAP and the prognosis of patients with AP.

Our study could not demonstrate that the CRP/ALB ratio is superior to the Ranson, MCTSI, and BISAP scores. The Ranson score is one of the earliest scores to assess the severity of AP that consists of 11 indicators that are assessed at admission and 48 h after admission (32). However, some of these indicators are not routinely collected during the early stages of AP, making early prediction difficult. The BISAP score that was introduced in 2008, consists of five indicators to predict AP mortality within 24 h of admission, which is simple to use, however, has low SAP predictive sensitivity (33, 34). In 2004, Mortele et al. formulated the MCTSI including a simplified evaluation of peripancreatic inflammation and the extent of pancreatic parenchymal necrosis and incorporated the extrapancreatic complications (vascular, gastrointestinal, and extrapancreatic parenchymal complications as well as the presence of pleural effusion and/or ascites) in the assessment (35). These indicators are closely related to the prognostic indicators of AP patients. Nevertheless, the calculation of the MCTSI score still requires the use of numerous parameters. A clinical course of AP greatly varies between patients and this makes the accurate classification and prediction of disease severity very important for both clinical decision-making and research recruitment (36). Our study demonstrated the predictive value of CRP/ALB in predicting SAP. The easily calculated CRP/ALB ratio may allow the estimation of the risk of SAP and adverse prognosis outcomes of AP, providing additional information that may facilitate the estimation of a patient's overall condition.

This study provides a simpler and more feasible tool for assessing the severity and prognosis of patients with AP. CRP/ALB value can help identify high-risk patients in advance and remind medical staff to strengthen ward care for high-risk patients. The CRP/ALB value can also distinguish MAP from SAP, and the results of this study can provide a reference for hospitals and health decision makers to develop more effective treatment strategies for AP gradient. However, the current study had some limitations. Firstly, it was a single-center retrospective study with a small number of cases included, which may limit the prognostic value of the CRP/ALB ratio. Secondly, we did not find an association between CRP/ALB ratio on day 1 and death and organ failure in AP patients. Thirdly, the lack of external verification in this study may limit the generalization of our results. Finally, from our findings, the CRP/ALB ratio may not be better than Ranson, MCTSI, and BISAP scores. The predictive value of the CRP/ALB ratio on SAP and the prognosis of AP needs to be further clarified.

## Conclusions

CRP/ALB ratio could predict SAP, death, pancreatic necrosis, and organ failure for AP, which may be a supplementary tool for the evaluations of SAP and prognosis for AP patients.

## Data Availability

The datasets used and/or analyzed during the current study are available from the corresponding author on reasonable request.
